# Editorial: Frontiers in dental medicine: Highlights in dental materials 2021/22

**DOI:** 10.3389/fdmed.2023.1152825

**Published:** 2023-02-20

**Authors:** Josette Camilleri

**Affiliations:** School of Dentistry, Institute of Clinical Sciences, University of Birmingham, Birmingham, United Kingdom

**Keywords:** material highlights, caries management, enamel bonding, implant coatings, aerosols, antibiobilm

**Editorial on the Research Topic** Frontiers in dental medicine: Highlights in dental materials 2021/22

The collection Highlights in Dental Materials 2021/22 was put together to showcase a selection of high-impact articles authored by leaders in the field. The work presented here highlights the broad diversity of research performed across articles published in the Dental Materials section, and the aim was to put a spotlight on the main areas of interest. The collection was skillfully edited by Prof Ziad Al-Dwairi from the Jordan University of Science and Technology.

This collection includes two original research articles, two brief research reports, and a review. The topics covered include the susceptibility of teeth to develop caries irrespective of the restorative material used, assessment of a nano-hydroxyapatite for caries management, enamel bonding, assessment of implant coating materials, and droplet spread in the clinical environment.

Caries research is key as dental caries is still a prevalent disease and is one of the main reasons for the loss of dental hard tissues, thus necessitating the use of dental materials. Secondary caries imposes a disease burden, as it results in the need to redo the restoration at a cost to the patient and requires further intervention and further movement along the restorative cycle of a tooth. In the study undertaken on patients attending the University of Pittsburgh School of Dental Medicine (as part of the Dental Registry and DNA Repository project), patients with secondary caries were identified and matched with patients with similar restorations that had not failed. Genomic DNA extracted from saliva was used to obtain genotypes in five markers of *MMP2* and *MMP3*. The results obtained in this study demonstrate the role of MMP2 in failure of restorations due to secondary caries independent of the restorative material. This important research has been published by Benli et al. in an article entitled “Matrix metalloproteinase 2 is associated with secondary caries independent from the restorative material”. This research clearly provides proof that secondary caries is not material-dependent, as has been previously postulated.

Another paper focusing on caries research and material interactions is the publication by Abo El-Gar et al. entitled “Potent antibacterial and antibiofilm activities of a synthetic remineralizing preparation of nano-hydroxyapatite against cariogenic Streptococcus mutans using an ex-vivo animal model”. This original research article investigates the use of a nano-hydroxyapatite in contact with bovine teeth and provides an evaluation of the bacterial inhibitory effect of the material, including the living tissue in this assessment. The use of tooth structure in the assessment of biological characteristics is important, as this leads to better clinical translation of the *in vitro* findings. The nanohydroxyapatite suspensions exhibited potential for remineralization and also exhibited antibiofilm characteristics.

Another contribution to this collection is the review “Update on enamel bonding strategies” by Sato et al. This is a very timely review article highlighting the differences between enamel and dentine bonding and discussing the advances made in this field. This review is particularly helpful for clinicians, enabling them to choose their clinical strategy carefully based on scientific evidence.

In an original scientific article on implant coatings by Shokeen et al. entitled “Surface characterization and assessment of biofilm formation on two titanium-based implant coating materials,” the authors investigate titanium nitride and titanium carbon nitride coatings over titanium implants. The surfaces were profiled and characterized, and it was shown that, in contrast with previous findings, there was no correlation between the surface roughness and hydrophobicity. The titanium carbon coating was hydrophilic and exhibited less microbial attachment when a well-established saliva-based oral microbial biofilm model was used, with growth conditions including relevant host components such as blood as well as the presence or absence of dietary carbohydrates.

A brief research report by Tsoi et al. “The spread of droplets and aerosols of surgical motor handpiece irrigation using different suction systems,” reports on their investigation of the clinical environment with regard to aerosol spread. Aerosols have been the subject of much discussion during the COVID pandemic and in the post-pandemic period. Although spread of aerosols is a constant clinical problem. This research shows that both external oral suction and high-volume suction are effective in reducing aerosols and droplets generated by the irrigation of a surgical high-speed motor handpiece.

These articles, all of which are published under the Research Topic Highlights in Dental Materials 2021/22, enrich our understanding of research in materials and its clinical translation. These publications break down barriers in relation to some of our beliefs that are based on practice rather than on scientific evidence. I wholeheartedly thank all the contributors and also the editorial team for putting together such an interesting collection.

